# Fully Automatic Fall Risk Assessment Based on a Fast Mobility Test

**DOI:** 10.3390/s21041338

**Published:** 2021-02-13

**Authors:** Wojciech Tylman, Rafał Kotas, Marek Kamiński, Paweł Marciniak, Sebastian Woźniak, Jan Napieralski, Bartosz Sakowicz, Magdalena Janc, Magdalena Józefowicz-Korczyńska, Ewa Zamysłowska-Szmytke

**Affiliations:** 1 Department of Microelectronics and Computer Science, Lodz University of Technology, 90-924 Lodz, Poland; rafal.kotas@p.lodz.pl (R.K.); marek.kaminski@p.lodz.pl (M.K.); pawel.marciniak@p.lodz.pl (P.M.); sebastian.wozniak@dokt.p.lodz.pl (S.W.); jan.napieralski@dokt.p.lodz.pl (J.N.); bartosz.sakowicz@p.lodz.pl (B.S.); 2 Audiology and Phoniatrics Clinic, Nofer Institute of Occupational Medicine, 91-348 Lodz, Poland; magdalena.janc@imp.lodz.pl (M.J.); zamysewa@imp.lodz.pl (E.Z.-S.); 3 Balance Disorders Unit, Department of Otolaryngology, Medical University of Lodz, 90-153 Lodz, Poland; magdalena.jozefowicz-korczynska@umed.lodz.pl

**Keywords:** bioinformatics, fall risk assessment, microsensors, decision support systems

## Abstract

This paper presents a fall risk assessment approach based on a fast mobility test, automatically evaluated using a low-cost, scalable system for the recording and analysis of body movement. This mobility test has never before been investigated as a sole source of data for fall risk assessment. It can be performed in a very limited space and needs only minimal additional equipment, yet provides large amounts of information, as the presented system can obtain much more data than traditional observation by capturing minute details regarding body movement. The readings are provided wirelessly by one to seven low-cost micro-electro-mechanical inertial measurement units attached to the subject’s body segments. Combined with a body model, these allow segment rotations and translations to be computed and for body movements to be recreated in software. The subject can then be automatically classified by an artificial neural network based on selected values in the test, and those with an elevated risk of falls can be identified. Results obtained from a group of 40 subjects of various ages, both healthy volunteers and patients with vestibular system impairment, are presented to demonstrate the combined capabilities of the test and system. Labelling of subjects as fallers and non-fallers was performed using an objective and precise sensory organization test; it is an important novelty as this approach to subject labelling has never before been used in the design and evaluation of fall risk assessment systems. The findings show a true-positive ratio of 85% and true-negative ratio of 63% for classifying subjects as fallers or non-fallers using the introduced fast mobility test, which are noticeably better than those obtained for the long-established Timed Up and Go test.

## 1. Introduction

Mobility dysfunctions present a serious problem in today’s ageing society. They result from several unrelated causes, including age-related muscle weakness and overall low endurance, diseases of the peripheral or central vestibular and musculoskeletal systems, and white matter lesions [1,2,3]. In addition, as some of these problems also occur in younger people, imbalance complaints are not restricted to the elderly [4].

One of the greatest risks faced by those with imbalance complaints is that of falling. Falls may result in dangerous injures, including bone fractures, to which the elderly are particularly prone. Moreover, in the case of elderly people living alone, a fallen person may not be able to stand up.

Considering the above, there is a need for objective tools to evaluate such mobility dysfunctions, including those than can assess the risk of falling. Any patients found to be at high risk may be diagnosed and treated for underlying diseases, an appropriate rehabilitation program may be introduced, or changes in their living surroundings may be made. It is also important to design objective tools for assessing such patients to track their rehabilitation progress.

Although there is already a significant accumulation of research concerning novel tools for fall risk assessment, which use capabilities of contemporary hardware and software solutions, none of the proposed approaches gained enough acceptance to replace long-established tests, such as the Timed Up and Go test (TUG) [1], Berg Balance Score (BBS) [2], Tinetti Test (TT) [3], or Dynamic Gait Index (DGI) [4]. This indicates that further research in this field is necessary.

A number of studies have examined the potential of inertial measurement units as measurement elements in fall risk assessment tools. A systematic review of 55 reported approaches developed for classification of subjects into fallers and non-fallers, as presented in [5,6,7], revealed accuracy in the range of 62–100%, sensitivity in the range of 55–100%, and specificity in the range of 35–100%. This means that at least some of the approaches provided satisfactory performance; however, further analysis reveals that the studies themselves have some methodological problems. Moreover, they are surprisingly restricted in their choice of the mobility tests performed by the subject, not investigating possibilities offered by alternatives. These shortcomings are further discussed below; the important novelty of the approach presented in this article is that it aims to avoid these pitfalls.

The most important problem identified in the previous studies is the approach towards labelling of the subjects. In order to construct a classifier, it is necessary to label all subjects in the study group as fallers or non-fallers beforehand; for this, a reliable and objective method must be used, as mislabelling the subjects nullifies all subsequent research. Of the investigated studies, 31% used prior fall history, 22% used prospective falls (i.e., reported after the test), and 29% used clinical assessment, while 18% of studies used some combination of the abovementioned methods. Unfortunately, all of these methods lack objectivity and are prone to misjudgement. In the case of the prior or prospective falls, the main obstacle is the fact that the subjects may have different daily routines (e.g., staying indoors or going out), vastly different home arrangements, and a varied approach towards activities subjectively perceived as risky; their choice of footwear may also influence the risk of falls. Moreover, as in most cases the falls are self-reported by the subjects (using a questionnaire), they may accidentally or purposefully enter inaccurate data. Even more problematic are the tests used during the clinical assessment: as they are themselves imperfect predictors of falls [8,9,10], any studies based on them simply duplicate the results of the clinical test rather than classify the subjects as fallers and non-fallers. Interestingly, the studies that report the best results are usually based on clinical assessment, not fall history (e.g., [11,12]). The study presented in this article uses the Sensory Organization Test (SOT) based on Computerized Dynamic Posturography (NeuroCom), which allows for objective assessment of fall susceptibility in a controlled environment, identical for all subjects. No previous studies have employed such an approach.

Another methodology problem, found in almost 50% of the investigated studies, is that they do not include any verification procedure for confirming the capabilities of the classifier. Data for all subjects are used to construct the classifier, with no cases set aside to check the classifier performance. This is a serious gap in the methodology, as it gives no indication of how accurately the model will perform in practice, when it will be used to classify cases that were not included in the learning set. The study presented in this article uses rigorous leave-one-out cross-validation to verify the model.

A further shortcoming of the previous approaches is that they mostly consider the same mobility tests (i.e., the tasks that the subjects are asked to perform). Of the investigated studies, 48% used walking, while 32% used the Timed Up and Go test. This means that there is very little research on new tests, which may prove to be more informative, or quicker to perform, or less cumbersome to the subjects. The study presented in this article uses a test that is a single element of the Berg Balance Score; it is very quick to perform and may be performed in a cramped space. It is also of interest as it does not contain a walking phase, so it gives an opportunity to investigate whether walking is necessary to assess fall risk. This test has not been investigated before as a sole indicator of fall risk.

The approach described in this study allowed a group of 40 subjects, comprising healthy subjects and those exhibiting the signs of central or peripheral vestibular dysfunction, to be classified as fallers and non-fallers with a true-positive ratio of 85% and true-negative ratio of 63%. The approach was based on the use of the described measurement and analysis approach on a task requiring the subject to transfer between chairs. Our findings compare favourably to those from the long-established Timed Up and Go task, applied to the same group and evaluated using time needed to complete the whole task and its phases.

## 2. Typical Approaches to Fall Risk Assessment

Quantitative balance assessment requires the use of dedicated devices such as a force plate. The most widely used method for such quantitative evaluations of balance deficit is static posturography, which analyses sways with the subject in a quiet stance. Static posturography quantifies the subject’s sways under four conditions: standing calm on a stable surface with eyes open and then eyes closed, and then standing on a foam surface with eyes open and then closed. Each condition is repeated three times for 10 s. Although static posturography may be useful in fall prediction [13,14,15], most relevant literature data concerns dynamic posturography [16]; this requires different devices, which are much more expensive and bulkier.

Other risk assessment tools are constantly being developed and verified. Not normalized functional testing involves a series of tasks, during which the patient performs simple but strictly determined operations such as standing motionlessly, walking, or sitting. Each task is evaluated by the physician through basic observation, possibly supported by simple tools such as a stopwatch or measuring tape. This situation has serious drawbacks: observation-based evaluation is difficult to quantify, may give different results from observer to observer (i.e., may lack objectivity), and is limited to features that can be observed using the naked eye.

Several clinical functional tests have been demonstrated to be of value in assessing the risk of falls. The Timed Up and Go (TUG) test is a popular test in which the patient is ordered to rise from a chair, walk three meters, turn around, walk back to the chair, and sit down. The time to complete the task is the output. The TUG test has been applied for assessing balance and gait deficits in Parkinson’s disease, multiple sclerosis, and stroke, as well as in other neurological diseases and orthopaedic disorders. It is used to evaluate the rehabilitation outcome and to predict the risk of falls in the elderly [17,18]. Unlike posturography, its evaluation requires only the simplest technical equipment: a stopwatch. As TUG is often regarded as the single most informative test for fall risk assessment [19,20,21], it will therefore be used as a benchmark for the new test.

The Berg Balance Scale (BBS) is an example of a score based on functional tests. It includes 14 simple tasks with a completion time of 20 min. These tests include sitting, standing with eyes open, picking up an object from the floor, and reaching, among others. The patient is rated on a five-point scale from zero to four based on whether the task was performed independently or with any help, protection, or support. The maximum number of points is 56. A score of 40–21 points indicates an average risk of falls, and values below 20 points indicate a high risk [2,22].

The Dynamic Gait Index (DGI) is a score based on eight tasks including gait with varying speed, gait with transverse and sagittal head movements, and gait over and around obstacles, among others. The tasks are rated on a four-point scale from zero to three. The maximum score is 24. A score less than 19 indicates a risk of falls [23,24].

Scores such as BBS or DGI are more detailed than single functional tests; however, they are lengthier and more inconvenient for patients.

## 3. Available Measurement and Analysis Solutions

Numerous technologies have been proposed for measuring human body movements. In most cases, the resulting devices are expensive and must be installed in the doctor’s office. The systems can be divided into the three following groups according to their mode of operation.

### 3.1. Approaches Based on Strain Gauges

Strain gauges quantify the forces acting on objects by measuring their resulting deformations. They are the technology of choice for posturography: centre-of-pressure movements on a measurement plate (e.g., those caused by a subject swaying), are measured by strain gauges near the corners. The resulting device is known as a force plate (or force platform). However, this technology limits the examination to a quiet stance or simple movements.

### 3.2. Approaches Based on MEMS Sensors

With the fast development of micro-electro-mechanical systems (MEMS), compact multi-sensor devices known as inertial measurement units (IMUs) have gained popularity in low-cost, wearable gait analysis systems. Two of the most important reasons why IMUs have gained such popularity is that the involved hardware is small and cheap. Several devices, such as BalanceFreedom™, SwayStar International™, and Vertiguard™, have been approved for use in Europe; in addition, some have been approved by the FDA for use within the U.S. as a real-time balance or rehabilitation tool (e.g., the Biodex Vibrotactile™ System [25]).

### 3.3. Other Approaches

Motion capture systems are commonly used to detect movements in the real world and transfer them to a virtual environment (VE). Various capture solutions exist based on different physical principles, such as optical, magnetic, and mechanical exoskeleton tracking systems. Dedicated camera-based solutions can track human movements extremely precisely and are sometimes used in professional sports training; however, their high cost and complex nature usually exclude them from everyday use. A virtual-reality-based exercise program has been found to offer promise for treating unilateral peripheral vestibular deficit [26]. Other more simplified approaches exist, including gaming systems such as the Microsoft Kinect.

Recently, motion tracking systems based on several IMUs have been introduced; such approaches allow body movements to be measured more precisely than single-sensor approaches, while still taking advantage of low-cost hardware. The solution presented in this paper is an example of such a system, tailored for the assessment of mobility dysfunctions.

## 4. Overview of the Solution

### 4.1. Intended Use

The solution presented in this article is intended for the assessment of mobility dysfunctions by physicians.

Unlike the force plate, the MEMS IMU allows data about the patient’s movements to be gathered during various tasks, as the patient is not limited in space during the examination. Several separate sensors may be used, which makes it possible to obtain information about individual body segments. The raw data can be subsequently processed by algorithms to precisely and objectively describe task performance. Objectivity is especially important for tracking the progress of the patient during rehabilitation, particularly when different doctors evaluate the patient’s mobility before and after therapy. However, this is also an important consideration when the same doctor evaluates the same patient over the course of three or six months: the assessment manner may change over time. In addition, the 3D rendering of a body model consisting of rigid segments by the software allows visualization of the patient’s body and its movements. This aids objective visual analysis, as the patient can be seen from different viewpoints, and the movements can be presented in slow motion. Finally, the data can also be saved for further reference or inclusion in the patient’s file.

### 4.2. Data Source

**Figure 1:**
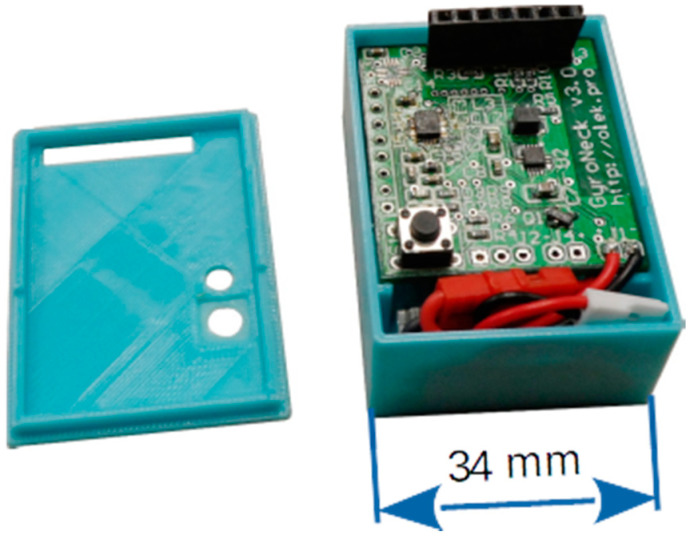
Measurement device.

The low cost of the proposed approach has been achieved by limiting the hardware part of the system to a minimum. Readings are obtained through small, battery-operated, Wi-Fi-enabled devices utilising the COTS IMU: three-axis accelerometer–gyroscope–magnetometer MEMS combo (Figure 1). The device is patent pending. Hardware considerations are not elaborated in this paper as they have little impact on the data processing paths; the reader is referred to the previous paper by the same authors [27]. Data storage and processing is provided by custom software running on a standard PC.

Scalability is possible: the data acquisition system has a flexible configuration, and various user-defined body models can be used. The results discussed in this article were obtained using a six-device configuration; however, a seven-device configuration has also been tested. The number of devices obviously affects both the accuracy and the overall cost of the solution.

**Figure 2:**
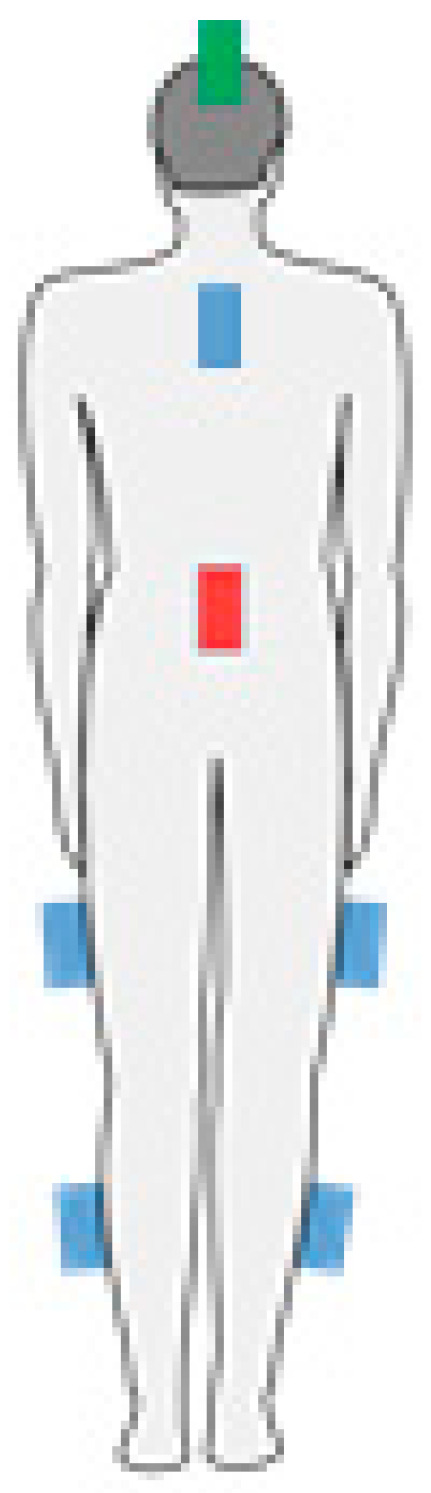
Placement of devices (coloured rectangles) on the body, various configurations: one-device—red; six-device—red and blue; seven-device—red, blue, and green.

The sensors are placed on the patient in such a way as to maximize the usefulness of the gathered information. The six-device configuration uses devices attached to the fifth lumbar vertebra (L5), cervico-thoracic transition (C7-TH1), thighs, and lower legs (Figure 2).

Most placement inaccuracies (tilt, rotation of the sensor) are dealt with through a calibration procedure, as explained in Section 5.3. However, while placement inaccuracies can be easily accounted for, problems can occur when the device changes its position relative to the body segment during the task (i.e., after calibration). For this reason, the attachment must be fixed securely; in the study, elastic bands were used for this purpose.

### 4.3. Processing Path

**Figure 3:**
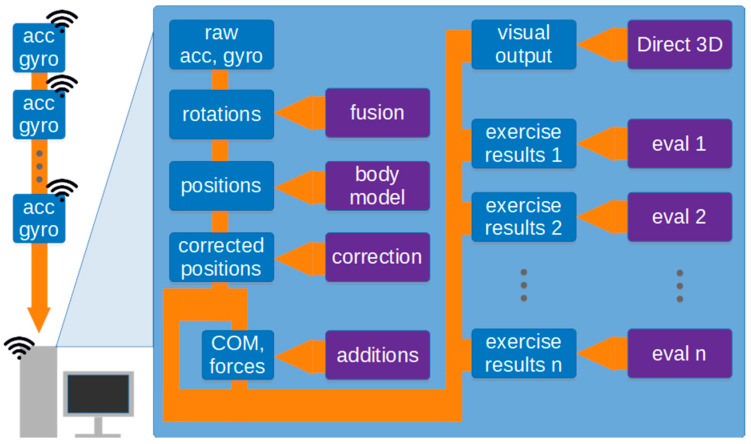
Outline of the processing path. Blue rectangles indicate data, purple rectangles indicate algorithms and models.

The processing path of the solution is outlined in Figure 3.

Data regarding individual body segment movements during the task are wirelessly transmitted to a PC, which stores the resulting time series and makes them available for analysis. These readings are then used to determine the rotations of body segments and, together with the body model, the complete body posture. Some supplemental information, such as position and movements of the centre of mass, or the forces acting on the ground, are calculated in the next step. The details of the algorithms employed in the whole process are discussed in Section 5.

The computed data is used, firstly, as a basis for visualization (see: Section 5.6) and, secondly, to quantitatively summarize the task via a collection of algorithms. One such task is the fast mobility test (see: Section 6).

## 5. Details of the Solution

### 5.1. Measurement Data

As outlined in Section 4.2, the solution may determine the orientation of body segments by using data from accelerometers, gyroscopes, and magnetometers. Each of these sensors has their own merits and drawbacks for the discussed application. For example, gyroscopes are well suited to recording object rotation but suffer from considerable drift. Accelerometers can be used to detect both the translation and rotation of an object but cannot distinguish between translation-related and gravity-related acceleration. The literature offers a number of approaches for combining such measurements. These include generic approaches, such as the Kalman filter [28], as well as other approaches, such as the Madgwick filter, which allow the computationally efficient fusion of IMU data [29]. As the Madgwick filter is also reported to have better accuracy than Kalman filter [30], it was selected for inclusion in the approach.

In addition, an algorithm that explicitly uses only accelerometer data for pitch and roll, and gyroscope data for yaw, has been implemented as an alternative approach. This approach ensures maximum pitch and roll accuracy to be achieved for stationary or quasi-stationary situations; this is essential for analysing some tasks performed by the patient, while still enabling yaw movement analysis.

### 5.2. Body Model

The body model allows computation of complete body posture and its change over time. The simplest body model can be built using data from only one IMU. In this case, the model consists of a single rigid segment. Its rotation can be determined by rotation data from the IMU, and the bottom of the segment remains affixed to a point on the ground. Despite its extreme simplicity, it is surprisingly useful for assessing some simple tasks (i.e., those based on stance and its variations).

**Figure 4:**
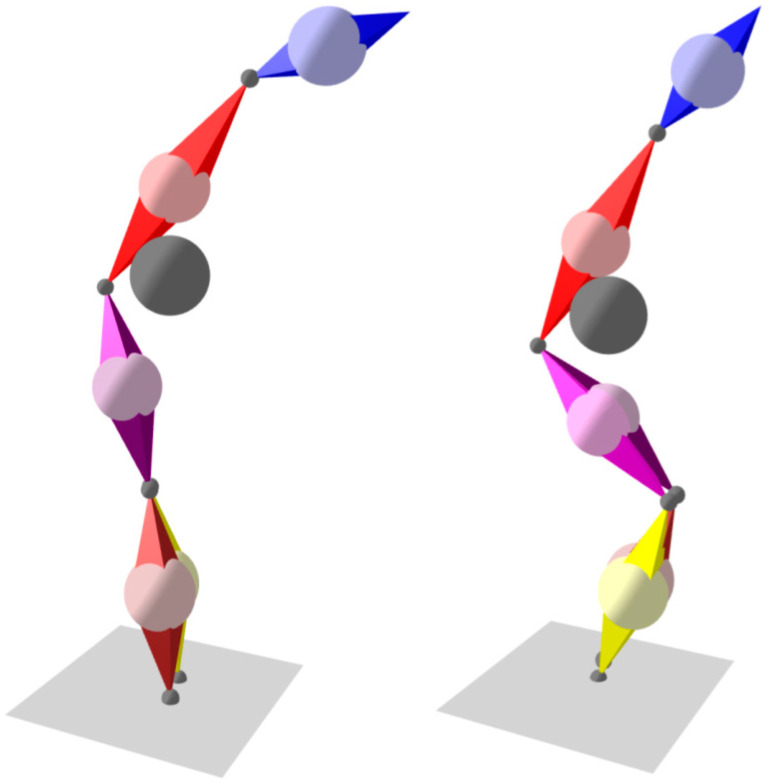
Calibration movements: forward lean and squat.

Further segments can be added to the model, and their rotations are determined by their corresponding IMUs. As the segments are connected through joints, if the translation of one segment is known (or assumed), together with its rotation and length, it can be used to determine the translations of the segments connected to it. This forms a chain of dependencies and allows a complete body posture to be computed. Both single-segment and six-segment models are presented in Figure 4. As no sensors are placed on the arms, their position is unknown and they are not included in the model; although this introduces some errors into the centre of mass calculation, the errors are small as arms only represent about 5% of total body mass [31].

The discussed solution does not use a hard-coded body model; instead, its definition could be adjusted to the patient and is loaded from a file. This definition can be created or edited using a simple integrated tool (editor). For each segment, its length, centre of mass position, connections to other segments, and colour (for visualization purposes) can be defined.

### 5.3. Calibration

The purpose of the IMUs is to measure absolute rotations of body segments (i.e., those expressed in real-world coordinates). However, IMUs can only provide data on their own absolute pitch and roll, as obtained from the accelerometers; in theory, magnetometer data could be used to determine yaw, but they are usually not reliable enough. This data cannot be treated as pitch and roll of the body segment due to possible misalignment between the coordinate systems of the IMU and of the body segment. Hardware-based adjustment mechanisms cannot be incorporated into measurement devices as they would significantly increase their complexity, bulk, and cost.

These problems can be overcome using a two-stage calibration procedure. Firstly, the Z-axes of the IMU can be aligned with those of the body segments by taking measurements with the patient standing upright. Secondly, the direction of the X-axes of the body segments (i.e., the “front” direction) can be determined using a predefined sequence of body movements: the patient leans forward and then squats (partial squat is enough) with the knees touching (Figure 4). These two movements ensure that all segments of the body on which IMUs are placed rotate along the real-world XZ plane; as the Z-axes are already determined, the X-axes can be calculated.

The calibration sequence is taken twice: once at the beginning and again at the end of a task session. The results are stored and used to correct the readings obtained during the session. Experience from the sessions recorded so far suggests that the calibration sequence recorded at the end of the session is more accurate: even though the sensors are firmly attached to the body segments, some minute changes in their position can occur during the first movements of the patient. Consequently, the end-of-session calibration is normally used; however, when it is improperly performed or clearly inaccurate, the beginning-of-session calibration can be used.

### 5.4. Walk Translation

As the employed body model is based on rotation data (see: Section 5.2), data regarding the translation of the body as a whole is not readily available. Nevertheless, many of the tasks used to diagnose mobility dysfunctions can be evaluated. Some involve no translation (e.g., free-standing, sitting down), while for others, translation data is not required; for example, in the Timed Up and Go test (see: Section 2), only the duration of the task and its phases are of interest, and the pivotal time instants can be determined solely based on segment rotations. However, translation is a valuable parameter in the case of walking, allowing the software to plot the trajectory of the centre of mass.

Although translation can be easily obtained from coordinate acceleration, MEMS accelerometers may not be precise enough for this purpose, especially in the case of slow-moving elderly persons (see also Section 5.1). Consequently, another method had to be employed. This alternative approach is based on the fact that a foot does not typically experience translation when in contact with the ground during walking; therefore, in theory, a foot can be considered an anchor point when on the ground. Unfortunately, this approach will not work in situations when the patient shuffles (i.e., walks without noticeably lifting the feet above the ground). To overcome this difficulty, another approach is added that determines the change in position of each foot in the direction of walking (indicated by the front direction of the torso). The change is computed relative to the middle point between the feet, with the foot that moves “backward” more prominently assumed to be the anchor point. This approach was found to be reliable enough in a variety of typical walks, although it will fail if someone walks unnaturally (if the patient goose steps or moonwalks being exaggerated examples).

### 5.5. Centre of Mass and Force Calculation

In many tasks, it is necessary to evaluate the movements of the centre of mass (COM) of the body. Its position is calculated based on the COMs of all body segments as a weighted vector sum.

In some tasks, it is also important to measure the force exerted by each foot on the ground. In the described solution, these forces are computed based on the COM movements of the body. These movements can be used to compute the acceleration of the COM, to which the force is proportional. This force is divided between the feet based on geometric principles; if one foot is raised, the problem becomes trivial.

### 5.6. Visualization

An important capability of the presented solution is the data visualization. Although this area will not be elaborated upon, as it was not directly utilized in obtaining the results, the approach, based on the use of a 3D model animated in real time, allowed the designed algorithms to be evaluated and improved more effectively than by directly interpreting the data coming from the sensors. Such visualization has been used for deeper analysis of cases in which the automatic algorithms were not able to evaluate the task. Usually, visual inspection revealed the problem to be improper performance of the calibration task, which precluded further analysis. However, in some situations, the sensors were found to have been placed on the incorrect body segments; in these cases, the problem was solved by altering the configuration in the software to match the true sensor placement.

**Figure 5:**
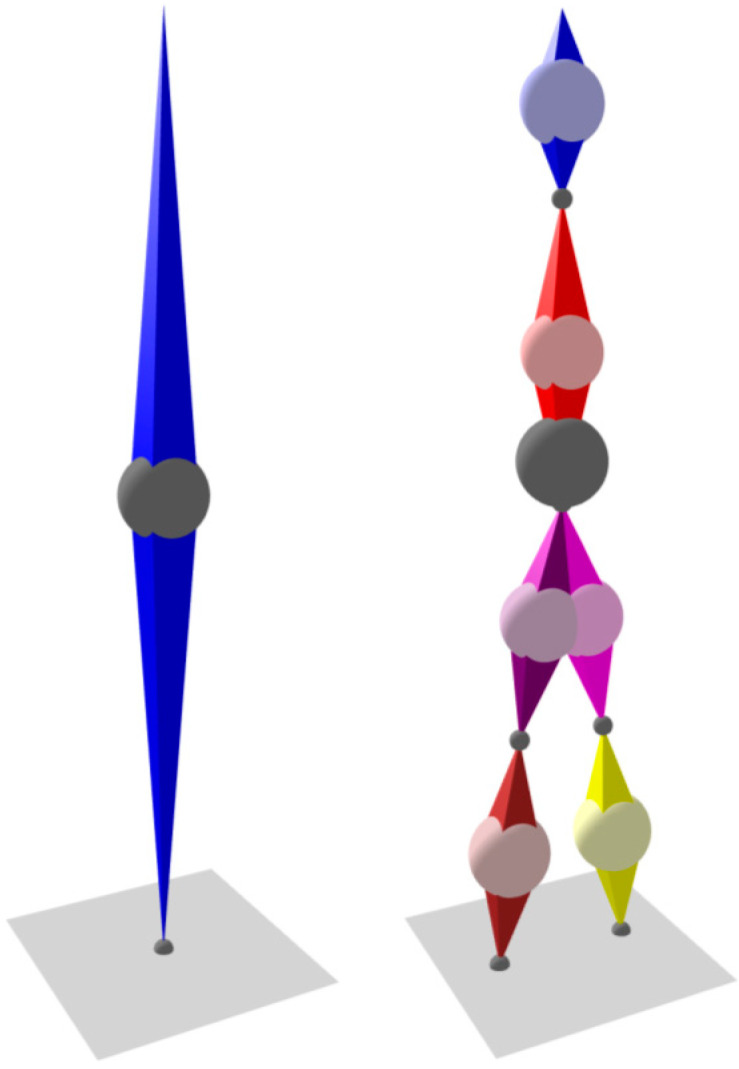
Single-segment and six-segment models. Balls indicate centre of mass: light colour—segment; dark grey—whole body.

Note that Figure 4 and Figure 5 are taken verbatim from the visualization pane of the solution’s PC software.

## 6. Swap Seats—Fast Test for Fall Risk Assessment

**Figure 6:**
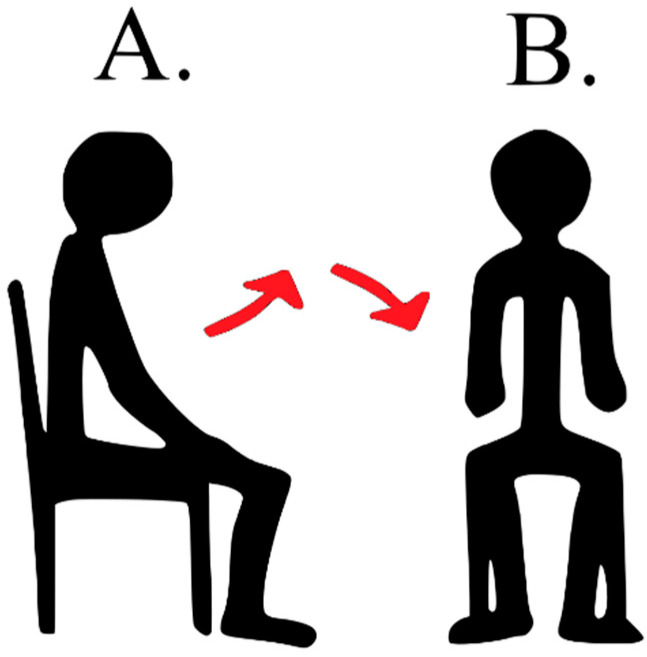
Visualization of the Swap Seats task (A—initial position, B—final position).

The Swap Seats test is originally an element of the BBS score. It was selected as it covers items important for functional assessment: balance, efficiency of the lower extremities, and the mobility possibilities of the subjects. It is also fast to perform (usually takes less than 15 s) and may be performed in a cramped floor space: a square with sides of 1.5 m is enough. During the test, the patient stands up from chair A, takes a step towards chair B, then turns the body 90 degrees and sits down in chair B. The distance between the chairs is one meter (see Figure 6). Chair A has no backrest, but the patient may use the backrest of chair B while sitting down. Clinically, the task is scored from zero to four points according to the ability to perform the task independently, or with any help, protection, and support.

The present study contrasts this test with the TUG test; the latter is also quick to perform but requires a 3 m walking distance.

Automatic evaluation of the Swap Seats task was performed in two forms (the number in the name indicates the number of computed features that characterise the performance of the task):

- SWAP 4—during the test, the time to perform the whole task was calculated (i.e., total duration), as well as the time needed to perform particular activities: viz. the duration of getting up and sitting down, as well as the time between the end of getting up and the beginning of sitting down (Table 1).

Although in theory it is possible to measure these times using a stopwatch, in practice it is difficult: as the times are short, even small measurement inaccuracies lead to large relative errors. In contrast, automatic evaluation using the described approach is straightforward and precise: it can detect the moment of rising from a chair and the moment of sitting back, thresholding segment rotations and their angular speeds. The algorithm was implemented as a state machine with the transitions between states triggered by exceeding the thresholds.

- SWAP 82—this approach combines the times from SWAP 4 with the maximum and minimum inclination angles of the head and the trunk while standing up and sitting down, as well as the angular speeds of the segments. The inclination angles are divided into two categories: the anterior–posterior inclination and the lateral inclination (Table 1). Note that measuring these angles and speeds is beyond the capabilities of a human observer, and therefore a measurement and computation system is required.

## 7. Experiment Setting and Methodology

The study group comprised 40 subjects who agreed to participate in the study and who had given their signed consent to participate. The study was approved by the Bioethics Committee. Fourteen subjects demonstrated signs of central or peripheral vestibular dysfunction, as indicated by detailed physical examination and Ulmer videonystagmography testing (saccades, smooth pursuit, optokinetic test, caloric test, rotational chair test).

**Figure 7:**
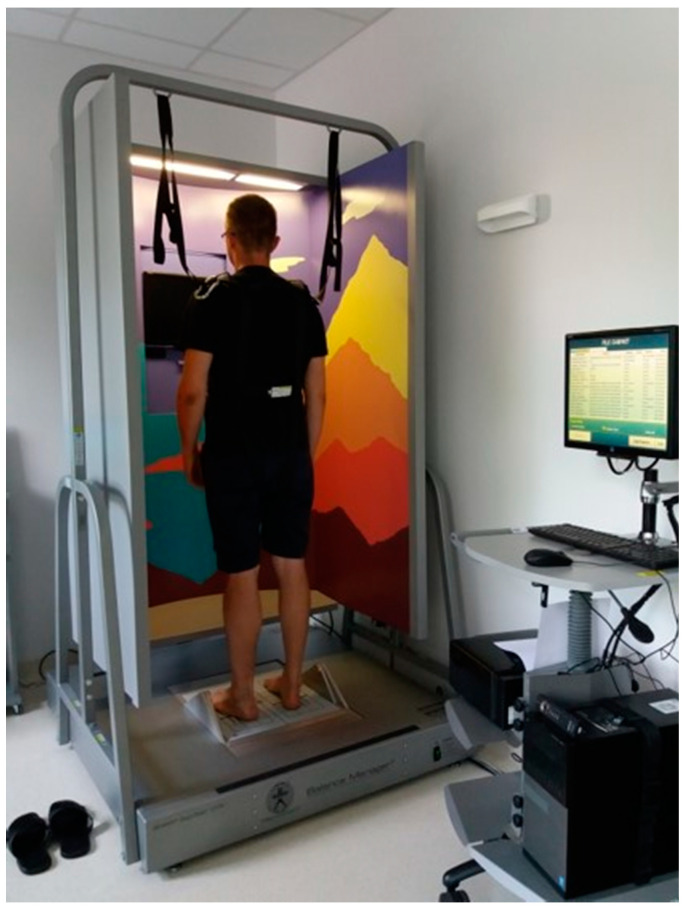
A subject undergoing the SOT test.

A point of particular interest in the present study was the susceptibility of patients to falls. In this study, classification into fallers and non-fallers was based on the falls observed during an examination performed with the Sensory Organization Test (SOT) based on Computerized Dynamic Posturography (NeuroCom). The test was performed using a dedicated device, consisting of a movable force plate positioned inside a movable booth (Figure 7). The test protocol consisted of six steps: 1. the eyes were open, and both the booth and the plate were stable; 2. the eyes were closed, and the plate was stable; 3. the eyes were open, and the plate was stable, but the booth swayed; 4. the eyes were open, and the plate swayed, but the booth was stable; 5. the eyes were closed, and the plate swayed; and 6. the eyes were open, and both the booth and the plate swayed. Each step lasted 20 s and was repeated three times. In order to ensure subjects’ safety, a loose harness was used to mitigate falls; it is designed so that the subject will not fall completely, yet it is possible to unambiguously identify fall occurrence. The SOT test allowed susceptibility to falls to be observed in a unified, controlled environment. The criterion of more than one fall during production of the SOT has been used as a fall predictor [32]. Falls were noticed for eight subjects from the group diagnosed with vestibular dysfunction. The study groups are summarized in Table 2.

After the SOT test, the subjects were asked to perform the SWAP test. To compare the performance of the proposed test with an existing approach, each subject was next asked to perform the TUG test. The kinetic tests (SWAP, TUG) were performed during the same session as the SOT test; this ensured that the subjects remained in the same psychophysical conditions during all tests.

In case of the TUG test, its evaluation was performed in a similar manner to that of SWAP 4: the duration of the whole test was measured automatically, as were the durations of its six component phases, hence the TUG 6 acronym used in this paper.

## 8. Results

The obtained data were used by the system for automatic classification of the subjects. For this purpose, the system employs a feed-forward artificial neural network (ANN). An ANN was chosen as the basic machine-learning tool as it can be easily used for both classification and regression; the latter being, however, beyond the scope of this article. The structure (number of layers, number of neurons in the hidden layer) and the activation functions can be chosen by the system operator. Supervised learning algorithms allow the ANN to be first trained on cases for which the classification output can be provided (e.g., doctor’s diagnosis, information about observed or past falls, etc.); following this, the trained ANN can be used on new cases.

The ANN used in the classifier follows the long-established pattern of a fully connected multi-layer perceptron. Although currently, more advanced structures are also being investigated, in particular deep learning models [33,34], in the current study the data obtained from the exercise are of a nature not suited for deep learning approaches. Deep learning shows its potential when used with raw data, as in [33], or when processing sequences (which can be sequences of raw data or sequences of features extracted from raw data, as in [34]). In the study presented in this article, the authors decided to compute features (raw data are not used), and the features are not organized in series; instead, they sum up the whole phase of the exercise (e.g., time of standing up or maximum angular speeds of segments during standing up). The authors intended to investigate how additional data available to the classifier influence its accuracy. For this, a reference had to be established. In this case, it is a classifier using only the data that could be acquired using basic technical equipment: durations of the phases of the exercise, which could be measured using a stopwatch (see TUG 6 and SWAP 4 below). Such a limited number of features makes deep learning structures superfluous. Consequently, the concept of features not organised in time series was also used for the classifier employing data available thanks to the IMU-based measurement system (see SWAP 82 below), in order to make the comparison with the reference fair, without introducing the additional factor of using a different type of the classifier. Note that fully connected multi-layer perceptrons are still being investigated as AI tools for fall risk assessment and remain among the best performing approaches [35].

The classification was performed with three different input sets, reflecting the tasks performed by the subject: (a) TUG 6, (b) SWAP 4, and (c) SWAP 82. For each set, an ANN with two hidden layers consisting of five, seven, and ten neurons with hyperbolic tangent activation function were designed. The output of the ANN was always the predicted class (faller, non-faller). Leave-one-out cross-validation was employed to ensure that the statistics are computed on samples not used during the ANN training. The results are presented in Table 3, Table 4, Table 5, Table 6, Table 7 and Table 8.

It should be stressed that the computation time of the introduced SWAP 82 procedure, using contemporary PC computers, is negligible: the classification decision is computed by the ANN in less than 10 µs when using a 4.8 GHz PC processor. Therefore, there is no penalty for using such an extensive number of features as input to the ANN.

## 9. Discussion

The TUG and SWAP tests include quite similar tasks: standing up, sitting down, and turning. The main differences are related to the walking task, which is not performed in the SWAP, and the rotation angle, which is 180 degrees in the TUG but 90 degrees in the SWAP. Consequently, SWAP is a simpler test and should therefore be less informative than TUG.

In this study, the TUG was analysed using more features than in the conventional test (viz. the times of each component phase versus total time). This was done in order to make the comparison with the SWAP test fairer, thus providing the ANN with more input to work with. Nevertheless, the TUG appears to offer mediocre predictive value regarding falls. This is a similar finding to that given in the literature [8]. Note that sensitivity was found to be higher than specificity in the present study, while most authors report higher specificity than sensitivity. However, similar values for Youden’s index (*J.*), a parameter that combines sensitivity and specificity (*J.* = sensitivity + specificity − 1), were obtained in the present study (*J.* = 0.12) as in previous studies (*J.* = 0.05) [36]. The SWAP test appears to be less informative (*J.* = 0.1); however, when more features are used as input values to the ANN, the specificity for this test increases dramatically, giving *J.* = 0.48.

The obtained results also suggest that walking is not an indispensable part of the test when the aim is to efficiently assess fall risk: unlike the TUG test, the SWAP test does not contain a walking phase, yet it outperforms TUG if analysed using a large number of features computed from the body movements. However, it has to be added that further investigation is needed to confirm these conclusions on a significantly broader group of subjects.

In constructing a classifier, it is important to find a combination of hyperparameters that allow both a good fit to the learning data and good generalisation properties. These two aims are contradictory. In particular, overfitting should be avoided, as it leads to seemingly good performance on the learning set, but poor performance on the test set. Overfitting can be avoided by limiting the size of the network, limiting learning duration, and using regularisation during learning. All of these approaches have been tested in the present study and verified using leave-one-out cross-validation. The analysis of the impact of ANN hyperparameters on the performance of the classifier reveals that a relatively small network is sufficient, even in the case of the SWAP 82 task: best performance for the SWAP 82 task was observed when seven neurons were included in the hidden layer, while even a smaller network with five neurons was best suited for SWAP 4 (Table 5). On the other hand, there was no need to limit the number of epochs during classifier learning (Table 6). L2 regularisation did not prove useful, at least for the tested network sizes—this is particularly visible for the SWAP 82 test (Table 7). The SWAP 82 test remains the best performer, regardless of the employed network structure.

In view of the results, it is tempting to further investigate the possibility of moving from lengthy and complicated tests designed for being evaluated using almost no technical equipment, such as the BBS, towards simple and fast tests evaluated using state-of-the-art technical equipment. This is particularly true considering that scales such as the BBS have been shown to be ineffective at predicting falls as a two-state classifier [9,10].

Constructing objective methods for detecting subjects at increased risk of falls is important as this allows for the introduction of procedures for fall prevention before actual falls happen. Therefore, one of the main aims of this study was to discriminate between fallers and non-fallers. However, a fall is not, strictly speaking, an illness; it is only a manifestation of various ailments that may have fundamentally different underlying causes. Hence it is difficult to construct “universal” classifiers, as the subjects who fall due to one illness may present vastly different movement patterns to those with others.

An important consideration is that recruitment for the study group was not restricted to persons with clearly defined illness. This could be considered a limitation of the study. On the other hand, the long-term aim of the authors is to provide a tool for evaluating fall risk before an illness is diagnosed. One possible approach to the problem of constructing a classifier based on a study group restricted to a single illness, yet capable of identifying mobility problems resulting from many illnesses, is to initially limit classifier learning to patients with a single, clearly defined illness, with the aim of eventually using a combination of such classifiers specialized for different illnesses. In addition to the abovementioned approach, further work will focus on constructing more advanced classifiers, for example convolutional neural networks, which have proven capable of discovering patterns in raw data.

Another limitation of the presented experiment is clearly the relatively small study group. Future studies will also aim to confirm the obtained results on larger databases.

## 10. Conclusions

Adding the inclination angles and angular speeds of body segments to the analysis significantly improves test performance. Among the tested group, the described approach based on data obtained from the SWAP 82 test allowed for noticeably better classification of fallers and non-fallers (when considered together) than the TUG 6 test: the findings were characterised by a true-positive ratio of 85% and a true-negative ratio of 63%. They are also better than those previously reported for the TUG test [8] and the BBS score [9,10], both considered as robust methods for evaluating patient mobility. This indicates that even very simple tests carry significant amounts of information and that such information can be extracted and effectively processed by the proposed measurement and analysis approach.

The obtained results offer comparable sensitivity, specificity, and accuracy to other solutions utilising IMU sensors, as referenced in the Introduction. However, unlike previous approaches, the present study attempted to label subjects as fallers and non-fallers based on an objective SOT test instead of fall history or clinical assessment; in addition, the classifier was also strictly tested through leave-one-out cross-validation.

The main contribution of this study was the aforementioned use of the SOT test, which has never been employed before in labelling the subjects prior to the construction of a fallers classifier, and also the investigation of a fast mobility test that has never before been used as sole data source for such a classifier.

## Figures and Tables

**Table 1:** Features computed during SWAP 4 and SWAP 82.

Feature ^a^	Unit
*total duration*	*s*
*raising duration*	*s*
*sitting duration*	*s*
*time between raising and sitting*	*s*
maximum A–P inclination of segment ^b^	deg
maximum L–R inclination of segment ^b^	deg
minimum and maximum inclination of segment ^c^	deg
average and maximum angular speed of segment ^c^	deg/s

^a^ SWAP 4 includes the features in italics, while SWAP 82 includes all features. ^b^ Separately for trunk and head segments, and for sitting and raising actions. ^c^ Separately for all segments and actions.

**Table 2:** Study group characteristics.

	Total	Fallers	Non-Fallers
Female	19	6	13
Male	21	2	19

**Table 3:** Sensitivity (true-positive rate, TPR), specificity (true-negative rate, TNR), and accuracy (ACC) for the faller classification using the TUG 6.

Neurons	Epochs	Regularisation	TUG 6		
			TPR	TNR	ACC
5	2	false	72%	38%	65%
5	2	true	81%	25%	70%
5	4	false	91%	13%	75%
5	4	true	97%	0%	78%
5	20	false	91%	63%	85%
5	20	true	100%	0%	80%
7	2	false	84%	25%	73%
7	2	true	75%	25%	65%
7	4	false	97%	13%	80%
7	4	true	97%	13%	80%
7	20	false	91%	50%	83%
7	20	true	100%	0%	80%
10	2	false	69%	50%	65%
10	2	true	57%	25%	50%
10	4	false	94%	25%	80%
10	4	true	97%	13%	80%
10	20	false	91%	50%	83%
10	20	true	100%	13%	83%

**Table 4:** Sensitivity (true-positive rate, TPR), specificity (true-negative rate, TNR), and accuracy (ACC) for the faller classification using the SWAP 4 and SWAP 82 tasks.

Neurons	Epochs	Regularisation	SWAP 4			SWAP 82		
			TPR	TNR	ACC	TPR	TNR	ACC
5	2	false	88%	75%	85%	94%	38%	83%
5	2	true	91%	25%	78%	88%	50%	80%
5	4	false	84%	38%	75%	84%	50%	78%
5	4	true	97%	0%	78%	81%	50%	75%
5	20	false	75%	38%	68%	88%	75%	85%
5	20	true	94%	13%	78%	81%	75%	80%
7	2	false	69%	38%	63%	91%	75%	88%
7	2	true	88%	25%	75%	78%	50%	73%
7	4	false	88%	13%	73%	82%	88%	83%
7	4	true	88%	0%	70%	88%	50%	80%
7	20	false	78%	38%	70%	91%	88%	90%
7	20	true	94%	0%	75%	88%	88%	88%
10	2	false	66%	63%	65%	88%	50%	80%
10	2	true	82%	25%	70%	78%	63%	75%
10	4	false	88%	25%	75%	88%	88%	88%
10	4	true	84%	13%	70%	81%	25%	70%
10	20	false	75%	25%	65%	84%	88%	85%
10	20	true	91%	13%	75%	81%	50%	75%

**Table 5:** Sensitivity (true-positive rate, TPR), specificity (true-negative rate, TNR), and accuracy (ACC) for the faller classification using the TUG 6, SWAP 4, and SWAP 82 tasks with different number of ANN neurons (averaged over number of epochs and regularisation approach).

	TUG 6			SWAP 4			SWAP 82		
	TPR	TNR	ACC	TPR	TNR	ACC	TPR	TNR	ACC
5 neurons	89%	23%	75%	88%	31%	77%	86%	56%	80%
7 neurons	91%	21%	77%	84%	19%	71%	86%	73%	83%
10 neurons	84%	29%	73%	81%	27%	70%	83%	60%	78%

**Table 6:** Sensitivity (true-positive rate, TPR), specificity (true-negative rate, TNR), and accuracy (ACC) for the faller classification using the TUG 6, SWAP 4, and SWAP 82 tasks with different number of epochs in ANN learning (averaged over number of neurons and regularisation approach).

	TUG 6			SWAP 4			SWAP 82		
	TPR	TNR	ACC	TPR	TNR	ACC	TPR	TNR	ACC
2 epochs	73%	31%	65%	80%	42%	73%	86%	54%	80%
4 epochs	95%	13%	79%	88%	15%	73%	84%	58%	79%
20 epochs	95%	29%	82%	84%	21%	72%	85%	77%	84%

**Table 7:** Sensitivity (true-positive rate, TPR), specificity (true-negative rate, TNR), and accuracy (ACC) for the faller classification using the TUG 6, SWAP 4, and SWAP 82 tasks with different regularisation approach in ANN learning (averaged over number of neurons and number of epochs).

	TUG 6			SWAP 4			SWAP 82		
	TPR	TNR	ACC	TPR	TNR	ACC	TPR	TNR	ACC
Without regularisation	87%	36%	76%	79%	43%	71%	88%	71%	84%
With regularisation	89%	13%	74%	90%	13%	74%	83%	56%	77%

**Table 8:** Sensitivity (true-positive rate, TPR), specificity (true-negative rate, TNR), accuracy, and Matthews correlation coefficient (MCC) for the faller classification using the TUG 6, SWAP 4, and SWAP 82 tasks. The outcomes are mean results for all network structures analysed in this study.

Parameter	TUG 6	SWAP 4	SWAP 82
TPR	88%	84%	85%
TNR	24%	26%	63%
Accuracy	75%	72%	81%
MCC	14%	10%	45%

## Data Availability

Data is not available due to privacy restrictions.
